# Molecular Phylogeny of Hantaviruses Harbored by Insectivorous Bats in Côte d’Ivoire and Vietnam 

**DOI:** 10.3390/v6051897

**Published:** 2014-04-29

**Authors:** Se Hun Gu, Burton K. Lim, Blaise Kadjo, Satoru Arai, Jeong-Ah Kim, Violaine Nicolas, Aude Lalis, Christiane Denys, Joseph A. Cook, Samuel R. Dominguez, Kathryn V. Holmes, Lela Urushadze, Ketevan Sidamonidze, Davit Putkaradze, Ivan V. Kuzmin, Michael Y. Kosoy, Jin-Won Song, Richard Yanagihara

**Affiliations:** 1 Pacific Center for Emerging Infectious Diseases Research, John A. Burns School of Medicine, University of Hawaii at Manoa, Honolulu, HI 96813, USA; E-Mail: sehungu@hawaii.edu; 2 Department of Natural History, Royal Ontario Museum, Toronto, ON M5S 2C6, Canada; E-Mail: burtonl@rom.on.ca; 3 Department of Biology, Université de Cocody, Abidjan 22, Côte d’Ivoire; E-Mail: blaisekadjo1@hotmail.com; 4 Infectious Disease Surveillance Center, National Institute of Infectious Diseases, Tokyo 162-8640, Japan; E-Mail: arais@nih.go.jp; 5 Department of Microbiology, College of Medicine, Korea University, Seoul 136-705, Korea; E-Mails: youminlove3@hotmail.com (J.-A.K.); jwsong@korea.ac.kr (J.-W.S.); 6 Departement Systematique et Evolution, UMR CNRS 7205, Muséum National d’Histoire Naturelle, Paris 75005, France; E-Mails: vnicolas@mnhn.fr (V.N.); lalis@mnhn.fr (A.L.); denys@mnhn.fr (C.D.); 7 Department of Biology, Museum of Southwestern Biology, University of New Mexico, Albuquerque, NM 87131, USA; E-Mail: cookjose@unm.edu; 8 Department of Pediatrics, School of Medicine, University of Colorado, Aurora, CO 80045, USA; E-Mails: samuel.dominguez@ucdenver.edu (S.R.D.); Kathryn.Holmes@ucdenver.edu (K.V.H.); 9 National Center for Disease Control and Public Health, Tbilisi 0177, Georgia; E-Mails: lelincdc@gmail.com (L.U.); keti_sida@yahoo.com (K.S.); dato.putkaradze@yahoo.com (D.P.); 10 Institute of Chemical Biology, Ilia State University, Tbilisi 0162, Georgia; E-Mail: lelincdc@gmail.com; 11 Global Alliance for Rabies Control, Manhattan, KS 66502, USA; E-Mail: ivkuzmin@yandex.ru; 12 Division of Vector Borne Diseases, Centers for Disease Control and Prevention, Fort Collins, CO 80521, USA; E-Mail: mck3@cdc.gov

**Keywords:** hantavirus, Chiroptera, evolution

## Abstract

The recent discovery of genetically distinct hantaviruses in multiple species of shrews and moles prompted a further exploration of their host diversification by analyzing frozen, ethanol-fixed and RNAlater^®^-preserved archival tissues and fecal samples from 533 bats (representing seven families, 28 genera and 53 species in the order Chiroptera), captured in Asia, Africa and the Americas in 1981–2012, using RT-PCR. Hantavirus RNA was detected in Pomona roundleaf bats (*Hipposideros pomona*) (family *Hipposideridae*), captured in Vietnam in 1997 and 1999, and in banana pipistrelles (*Neoromicia nanus*) (family *Vespertilionidae*), captured in Côte d’Ivoire in 2011. Phylogenetic analysis, based on the full-length S- and partial M- and L-segment sequences using maximum likelihood and Bayesian methods, demonstrated that the newfound hantaviruses formed highly divergent lineages, comprising other recently recognized bat-borne hantaviruses in Sierra Leone and China. The detection of bat-associated hantaviruses opens a new era in hantavirology and provides insights into their evolutionary origins.

## 1. Introduction

Hantaviruses (genus *Hantavirus*, family *Bunyaviridae*) possess a negative-sense, single-stranded, tripartite segmented RNA genome, consisting of large (L), medium (M) and small (S) segments, encoding an RNA-dependent RNA polymerase (RdRp), envelope glycoproteins (Gn and Gc) and a nucleocapsid (N) protein, respectively [1]. To date, 23 hantaviruses, hosted by reservoir rodent species, have been recognized as distinct species by the International Committee on Taxonomy of Viruses [2]. Several of these rodent-borne hantaviruses cause acute, febrile diseases of varying clinical severity and lethality in humans, known as hemorrhagic fever with renal syndrome and hantavirus cardiopulmonary syndrome [3]. Though once believed to be restricted to rodents (order Rodentia, family *Muridae* and *Cricetidae*), the reservoir host range of hantaviruses is far more expansive, as evidenced by the detection of divergent lineages of hantaviruses in multiple species of shrews and moles (order Soricomorpha, family *Soricidae* and *Talpidae*) throughout Asia, Europe, Africa and North America [4,5,6,7,8,9,10,11,12,13,14,15,16,17,18,19]. 

Despite their phylogenetic relatedness to the European mole (*Talpa europaea*) within the Laurasiatheria [20,21], as well as their rich genetic diversity, vast geographic range and ability to host many disease-causing viruses [22,23,24], bats (order Chiroptera) have not been extensively studied as potential reservoirs of hantaviruses. Although serological evidence of hantavirus infection was reported in the common serotine (*Eptesicus serotinus*) and greater horseshoe bat (*Rhinolophus ferrumequinum*) captured in Korea [25], genetic analysis of hantavirus isolates from these bat species suggested laboratory contamination [26].

The genetic diversity of newfound hantaviruses recently detected in insectivorous bats preclude any possibility of contamination: Mouyassué virus (MOYV) in the banana pipistrelle (*Neoromicia nanus*) from Côte d’Ivoire [27]; Magboi virus (MGBV) in the hairy slit-faced bat (*Nycteris hispida*) from Sierra Leone [28]; Xuan Son virus (XSV) in the Pomona roundleaf bat (*Hipposideros pomona*) from Vietnam [29]; Huangpi virus (HUPV) in the Japanese house bat (*Pipistrellus abramus*) and Longquan virus (LQUV) in the Chinese horseshoe bat (*Rhinolophus sinicus*), Formosan lesser horseshoe bat (*Rhinolophus monoceros*) and intermediate horseshoe bat (*Rhinolophus affinis*) from China [30]. The primary goal of this multi-national collaborative study was to extend the search for hantaviruses in bats and to obtain more of the MOYV and XSV genomes. Our data indicate that bat-borne hantaviruses and Nova virus, a hantavirus hosted by the European mole, comprise a highly divergent phylogenetic lineage, suggesting that ancestral bats and/or soricomorphs, rather than rodents, may have served as the early reservoir hosts of primordial hantaviruses.

## 2. Results and Discussion

### 2.1. Hantavirus Detection and Sequence Analysis

**Figure 1:**
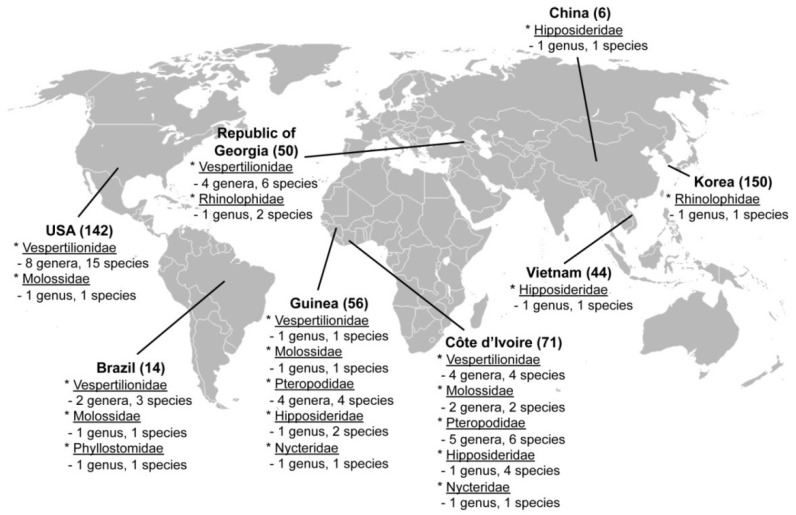
Geographic origin of 533 specimens from bats, belonging to seven families, were analyzed for hantavirus RNA, using RT-PCR. The number of samples and genera and species of bats are shown for each country.

**Figure 2:**
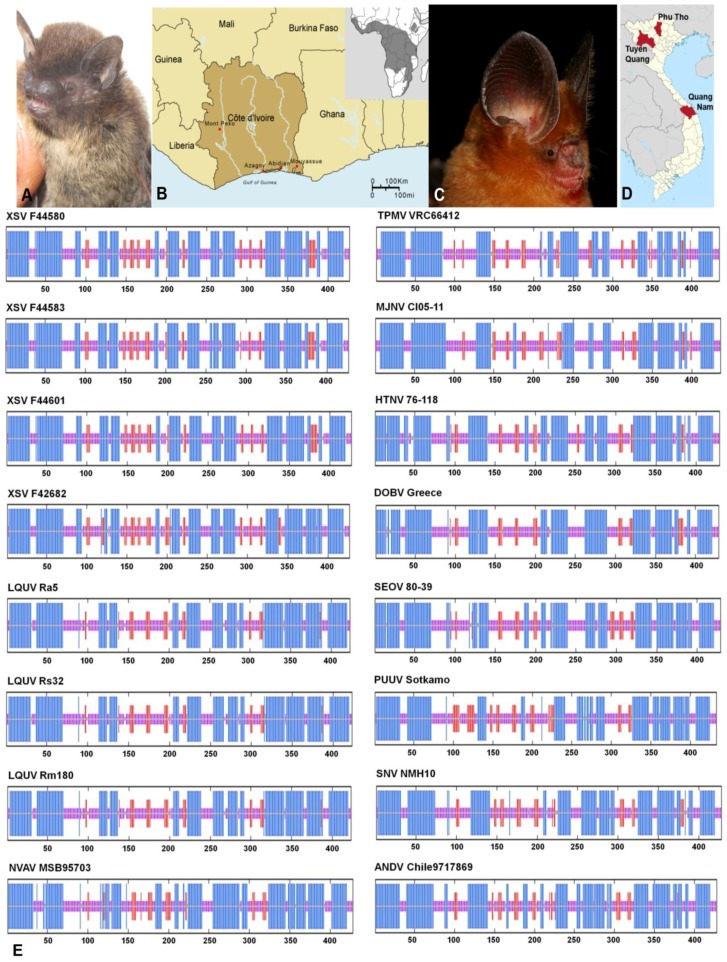
(**A**) Banana pipistrelle (*Neoromicia nanus*); (**B**) Map of Cote d’Ivoire, showing site where Mouyassué virus-infected banana pipistrelles were captured during June 2011; inset shows geographic distribution of banana pipistrelle; (**C**) Pomona roundleaf bat (*Hipposideros pomona*); (**D**) Map of Vietnam, showing Phu Tho, where Xuan Son virus (XSV) was first discovered, and Tuyên Quang and Quang Nam, where Pomona roundleaf bats were captured in May 1997 and March 1999, respectively; (**E**) Comparison of the consensus secondary structures of the nucleocapsid protein of XSV, Longquan virus (LQUV), Nova virus (NVAV), Thottapalayam virus (TPMV), Imjin virus (MJNV), Hantaan virus (HTNV), Dobrava virus (DOBV), Seoul virus (SEOV), Puumala virus (PUUV), Sin Nombre virus (SNV) and Andes virus (ANDV), as predicted using methods available on the NPS@ structure server [32]. Alpha helices are represented by blue bars, beta strands by red bars, and random coils and unclassified structures by magenta and gray bars, respectively.

Exhaustive attempts to detect hantaviruses were unsuccessful in nearly all of the 454 bat tissue samples (Table 1 and Figure 1), despite employing oligonucleotide primers and PCR cycling conditions used to find MOYV [27] and XSV [29]. In addition, hantavirus RNA was not detected in any of the 79 rectal swab and fecal samples. Because LQUV was previously found in four species of horseshoe bats in China [30], we expected to find the same or a similar hantavirus in the greater horseshoe bat, captured on Jeju Island in Korea. However, this was not the case, in spite of using LQUV-specific primers. Nevertheless, we did manage to obtain more of the MOYV and XSV genomes. That is, the original report of MOYV in the banana pipistrelle (Figure 2A,B) was based on a 423-nucleotide region of the L segment [27]. Through repeated trial-and-error efforts, suitable primers were designed to obtain an additional 1268 nucleotides of the L segment (Table 2). 

In addition, Arai and colleagues previously reported a novel hantavirus, designated XSV, in one of five Pomona roundleaf bats, captured during July 2012 in Xuan Son National Park in Phu Tho province in northern Vietnam [29]. In analyzing archival kidney tissues from 44 Pomona roundleaf bats trapped in Tuyên Quang and Quang Nam provinces, hantavirus L-segment sequences were detected in five animals (Figure 2C,D). Although a 15.7%–19.2% difference was found at the nucleotide level with prototype XSV, the high amino acid sequence similarity was consistent with these sequences representing genetic variants of XSV. Pair-wise alignment and comparison of the full-length S segment of XSV, amplified and sequenced from four bats (Table 2), indicated sequence similarity of 58.9%–60.3% at the amino acid level with LQUV, the only other bat-borne hantavirus for which the entire S segment has been sequenced. And sequence analysis of a 663-nucleotide (221 amino acid) region of the Gc envelope glycoprotein-encoding M segment showed that XSV differed by >45% from representative hantaviruses harbored by rodents and most soricomorphs. Collectively, the high level of sequence divergence in the N protein and Gc glycoprotein between XSV and other hantaviruses suggests that it might represent a new hantavirus species, using the guidelines proposed by Maes and co-workers [31]. However, the definitive taxonomic classification of XSV and other bat-borne hantaviruses must await their isolation in cell culture.

**Table 1:** Specimen types analyzed for hantavirus RNA.

Bat Family	Frozen			RNAlater®				Ethanol-fixed	Total
	Lung	Liver	Kidney	Lung	Intercostal Muscle	Intestine	Rectal Swab or Feces	Liver	
*Hipposideridae*			50	7					57
*Molossidae*	1			35			6		42
*Nycteridae*				1	1				2
*Pteropodidae*				42	18				60
*Phyllostomidae*	2								2
*Rhinolophidae*	150			12					162
*Vespertilionidae*	11	17	1	49		45	73	12	208
Total	164	17	51	146	19	45	79	12	533

**Table 2:** Xuan Son virus and Mouyassué virus in insectivorous bats.

Virus	Strain	Bat Species	Country	Province	S	M	L
XSV	VN1982	*Hipposideros pomona*	Vietnam	Phu Tho	499 bp		4582 bp
					KC688335		JX912953
	F42640			Tuyên Quang	516 bp		567 bp
					KF704708		KF704713
	F42682				1752 bp	663 bp	1160 bp
					KF704709	KJ000538	KF704714
	F44580			Quang Nam	1728 bp		804 bp
					KF704710		KF704715
	F44583				1728 bp		1160 bp
					KF704711		KF704716
	F44601				1728 bp	663 bp	1160 bp
					KF704712	KJ000539	KF704717
MOYV	KB576	*Neoromicia nanus*	Côte d'Ivoire	Mouyassué			1691 bp
							JQ287716
	KB577						372 bp KJ000540

### 2.2. Nucleocapsid Secondary Structure

In employing software available on the @NPS structure server [32], the overall predicted secondary structures of the N proteins were similar. That is, despite the relatively low amino acid sequence similarity among the rodent-, shrew-, mole- and bat-borne hantaviruses, the N protein comprised two major α-helical domains packed against a central β-pleated sheet (Figure 2E). However, the central β-pleated sheet motif of XSV, including the RNA-binding region (amino acid positions 175 to 217), was unlike that of other hantaviruses, even that of LQUV, which more closely resembled murid rodent-borne hantaviruses, such as Hantaan virus (HTNV 76-118), Dobrava virus (DOBV Greece) and Seoul virus (SEOV 80-39) (Figure 2E). The distinctive α-helix motif between two β-strands of the RNA-binding region, observed in the prototype mole-borne hantavirus, Nova virus (NVAV MSB95703), as well as HTNV and SEOV, but not in LQUV, may have a significant effect on binding specificity.

### 2.3. Phylogenetic Analysis

**Figure 3:**
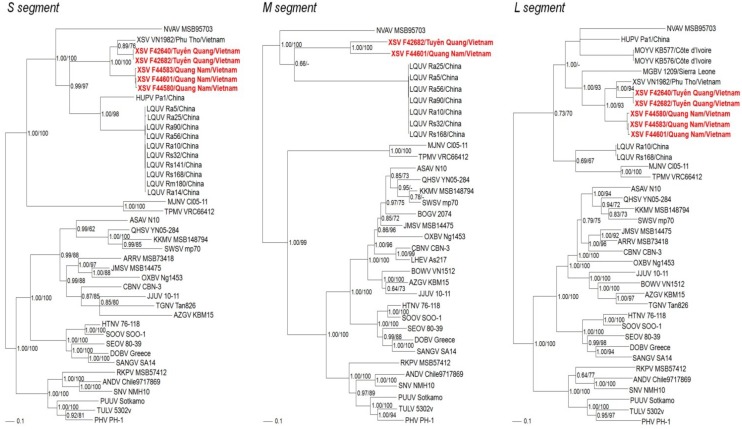
Phylogenetic trees were generated by maximum-likelihood and Bayesian methods, using the GTR+I+Γ model of evolution, based on the S-, M- and L-genomic sequences of hantavirus strains. Because tree topologies were nearly identical using RAxML and MrBayes programs, the trees generated by MrBayes were displayed. The evolutionary relationships between Xuan Son virus (XSV), Mouyassué virus (MOYV) and other bat-borne hantaviruses, including Magboi virus (MGBV), Longquan virus (LQUV)and Huangpi virus (HUPV), are shown, as are representative soricomorph-borne hantaviruses, including Nova virus (NVAV MSB95703, S: FJ539168; M: HQ840957; L: FJ593498), Thottapalayam virus (TPMV VRC66412, S: AY526097; M: EU001329; L: EU001330), Imjin virus (MJNV Cl05-11, S: EF641804; M: EF641798; L: EF641806), Seewis virus (SWSV mp70, S: EF636024; M: EF636025; L: EF636026), Kenkeme virus (KKMV MSB148794, S: GQ306148, M: GQ306149; L: GQ306150), Lianghe virus (LHEV As217, M: JX465406), Boginia virus (BOGV 2074, M: JX990966), Cao Bang virus (CBNV CBN-3, S: EF543524; M: EF543526; L: EF543525), Ash River virus (ARRV MSB 73418, S: EF650086; L: EF619961), Jemez Springs virus (JMSV MSB144475, S: FJ593499; M: FJ593500; L: FJ593501), Qian Hu Shan virus (QHSV YN05-284, S: GU566023; M: GU566022; L: GU566021), Tanganya virus (TGNV Tan826, S: EF050455; L: EF050454), Azagny virus (AZGV KBM15, S: JF276226; M: JF276227; L: JF276228), Jeju virus (JJUV 10-11, S: HQ834695; M: HQ834696; L: HQ834697), Bowé virus (BOWV VN1512, M: KC631783; L: KC631784), Asama virus (ASAV N10, S: EU929072; M: EU929075; L: EU929078), Oxbow virus (OXBV Ng1453, S: FJ5339166; M: FJ539167; L: FJ593497) and Rockport virus (RKPV MSB57412, S: HM015223; M: HM015219; L: HM015221). Also shown are the phylogenetic positions of representative rodent-borne hantaviruses, including Hantaan virus (HTNV 76-118, S: NC_005218; M: Y00386; L: NC_005222), Soochong virus (SOOV SOO-1, S: AY675349; M: AY675353; L: DQ056292), Dobrava virus (DOBV Greece, S: NC_005233; M: NC_005234L: NC_005235), Seoul virus (SEOV 80-39, S: NC_005236; M: NC_005237; L: NC_005238), Sangassou virus (SANG SA14, S: JQ082300; M: JQ082301; L: JQ082302),Tula virus (TULV M5302v, S: NC_005227; M: NC_005228; L: NC_005226), Puumala virus (PUUV Sotkamo, S: NC_005224; M: NC_005223; L: NC_005225), Prospect Hill virus (PHV PH-1, S: Z49098; M: X55129; L: EF646763), Sin Nombre virus (SNV NMH10, S: NC_005216; M: NC_005215; L: NC_005217) and Andes virus (ANDV Chile9717869, S: NC_003466; M: NC_003467; L: NC_003468). The numbers at each node are posterior node probabilities (left) based on 150,000 trees and bootstrap values (right) based 1000 replicates executed on the RAxML BlackBox web server, respectively. The scale bars indicate nucleotide substitutions per site.

Phylogenetic analyses, based on S-, M- and L-genomic sequences, indicated that XSV and MOYV shared a common ancestry with other bat-borne hantaviruses (Figure 3). In all analyses, NVAV from the European mole segregated with the bat-associated hantaviruses, which was reminiscent of trees based on the complete mitochondrial genomes of the European mole and bats [20,21]. The basal position of chiropteran-borne hantaviruses and selected soricomorph-borne hantaviruses, such as Nova virus in the European mole, Thottapalayam virus in the Asian house shrew and Imjin virus in the Ussuri white-toothed shrew, in phylogenetic trees based on the S- and L-genomic sequences suggests that soricomorphs and/or chiropterans, rather than rodents, may have been the primordial mammalian hosts of ancestral hantaviruses (Figure 3). Geographic-specific clustering was evidenced by the close phylogenetic relationship between prototype XSV VN1982 from Phu Tho province and XSV F42640 and XSV F42682 from neighboring Tuyên Quang province in northern Vietnam. On the other hand, XSV F44583, XSV 44601 and XSV 44580 from Quang Nam province in central Vietnam clustered together. Although limited differences were present in phylogenetic trees based on each segment, tree topologies were generally congruent and supported by significant bootstrap values (>70%) and posterior node probabilities (>0.70). 

### 2.4. Bats as Hosts of Hantaviruses

The phylogeny of bats is not fully resolved [21]. The order Chiroptera was traditionally divided in two suborders, Megachiroptera and Microchiroptera. However, due to the paraphyly of the Microchiroptera, a new taxonomic nomenclature, comprising the suborder Yinpterochiroptera (megabats or fruit bats in the Pteropodidae family in Megachiroptera and a few Microchiroptera families) and Yangochiroptera (the remaining Microchiroptera families), has been proposed [33]. In the former classification, bat species hosting hantaviruses belong only to the Microchiroptera suborder, but in the Yinpterochiroptera-Yangochiroptera classification, they belong to both suborders, suggesting that primordial hantaviruses may have emerged in an early common ancestor of bats. 

Within the Microchiroptera, hantaviruses are found in bats belonging to four phylogenetically distant families, namely *Hipposideridae* (Old World leaf-nosed bats) and *Rhinolophidae* (horseshoe bats) in the suborder Yinpterochiroptera, and *Nycteridae* (hollow-faced bats) and *Vespertilionidae* (vesper bats) in the suborder Yangochiroptera. The families *Hipposideridae* and *Vespertilionidae* are among the most speciose insectivorous bats, with member species distributed across Africa, Europe, Asia, the Americas and Australia. Their vast geographic distribution provides unlimited opportunities to search for related bat-associated hantaviruses.

Compared to the multitude of hantaviruses reported from approximately 50% of soricomorph species tested [34,35], the cumulative number of newly recognized bat-borne hantaviruses is exceedingly low, if one considers the 533 bat samples tested in the present study, along with the nearly 1200 bat specimens analyzed in four other studies [27,28,29,30]. The modest proportion of hantavirus RNA detection in bat tissues may be attributed to the highly divergent nature of their genomes, as well as the very focal or localized nature of hantavirus infection, small sample sizes of bat species, primer mismatches, suboptimal PCR cycling conditions, and variable tissue preservation with degraded RNA [27,29]. Alternatively, bats may be less susceptible to hantavirus infection or may have developed immune mechanisms to curtail viral replication and/or persistence. For answers to such questions, and myriad others, reagents need to be developed and multidisciplinary collaborative studies must be designed to collect optimal specimens to isolate and characterize these newfound bat-borne hantaviruses. Only then will a better understanding be gained about their evolutionary origins and phylogeography, co-evolution history, transmission dynamics and pathogenic potential.

## 3. Experimental Section

### 3.1. Samples

Archival frozen, ethanol-fixed and RNAlater^®^-preserved tissues from bats, captured during 1981–2012 in Brazil, China, Cote d’Ivoire, Guinea, Korea, Republic of Georgia, Vietnam and the United States (Figure 1 and Table 1), were tested for hantavirus RNA by RT-PCR, using newly designed and previously employed oligonucleotide primers [12,18,27,29]. Of the 533 samples tested, the majority consisted of lung (310) and kidney (51) tissues (Table 1). RNA extracted from rectal swabs and feces (79) were also tested. Bats were from seven families (*Hipposideridae*, *Molossidae*, *Nycteridae*, *Pteropodidae*, *Phyllostomidae*, *Rhinolophidae* and *Vespertilionidae*), 28 genera and 53 species (Figure 1). The University of Hawaii Institutional Animal Care and Use Committee approved the use of archival tissues as being exempt from protocol review.

### 3.2. Genome Detection and Sequencing

Total RNA extraction from tissues, using the PureLink Micro-to-Midi total RNA purification kit (Invitrogen, San Diego, CA, USA), and cDNA synthesis, using the SuperScript III First-Strand Synthesis Systems (Invitrogen) with random hexamers, were performed as described previously [9,12,18]. Oligonucleotide primers used to amplify S-, M- and L-genomic segments of bat-borne hantaviruses are listed on Table 3. First- and second-round PCR were performed in 20-μL reaction mixtures, containing 250 μMdNTP, 2.5 mM MgCl_2_, 1 U of Takara LA Taq polymerase (Takara, Shiga, Japan) and 0.25 μM of each primer [16]. Initial denaturation at 94 °C for 2 min was followed by two cycles each of denaturation at 94 °C for 30 s, two-degree step-down annealing from 46 °C to 38 °C for 40 s, and elongation at 72 °C for 1 min, then 30 cycles of denaturation at 94 °C for 30 s, annealing at 42 °C for 40 s, and elongation at 72 °C for 1 min, in a GeneAmp PCR 9700 thermal cycler (Perkin-Elmer, Waltham, MA, USA) [6,9,11,12,16]. PCR products, separated using MobiSpin S-400 spin columns (MoBiTec, Goettingen, Germany), were sequenced directly using an ABI Prism 3130 Genetic Analyzer (Applied Biosystems, Foster City, CA, USA) [9,16]. 

**Table 3:** Oligonucleotide primers used to amplify Xuan Son virus and Mouyassue virus from insectivorous bat tissues.

Primer	Sequence (5’-3’)	Segment	Polarity
Han-5’end-EcoRI	CTC GAA TTC TAG TAG TAG AC	S	+
Shrew-S777R	AAN CCD ATN ACN CCC AT	S	-
Shrew-S764R	CCA TNA CWG GRC TNA TCA	S	-
XSV-S627F	AGA AGA ATT GAC ACC TGG GCG AT	S	+
XSV-S1040F	CAT TCT TTT CAC TGT TGC AGG A	S	+
XSV-S1235R	GTT CTT CTG AGA TAT GAC TGA TA	S	-
Bat-3’endR	TAG TAG TAK RCT CCC T	S	-
G2F1	TGG GCT GCA AGT GC	M	+
Han-M2957R	GAR CCC CAN GCN CCN TCW AT	M	-
Han-M2631R	CAT NAY RTC NCC RGG RTC NCC	M	-
Han-L1880F	CAR AAR ATG AAR NTN TGT GC	L	+
Bat-L1929F	ATG AAR NTN TGT GCA YTG TTT GA	L	+
Han-L2520F	ATN WGH YTD AAR GGN ATG TCN GG	L	+
Bat-L2810F	GAR GAY TAY TAT GAT G	L	+
Han-L3000R	GCN GAR TTR TCN CCN GGN GAC CA	L	-
Han-L2970R	CCN GGN GAC CAY TTN GTD GCA TC	L	-
MOYV-L2683R	GCT GGA TAA CAG TCG GGT TTA ATC	L	-
MOYV-L2612R	TAA GTG CCC ATC TTC TTG TA	L	-
Bat-L3442R	ACC ART CWG AMC CAT CAT C	L	-
Bat-L3613R	GTA GAG AGA AAC TCT GCA TTT GT	L	-

### 3.3. Phylogenetic Analysis

Maximum likelihood and Bayesian methods, implemented in RAxML Blackbox webserver [36] and MrBayes 3.1 [37], under the best-fit GTR+I+Γ model of evolution [38] and jModelTest version 0.1 [39], were used to generate phylogenetic trees. Two replicate Bayesian Metropolis–Hastings Markov Chain Monte Carlo runs, each consisting of six chains of 10 million generations sampled every 100 generations with a burn-in of 25,000 (25%), resulted in 150,000 trees overall. The S, M and L segments were treated separately in phylogenetic analyses. Topologies were evaluated by bootstrap analysis of 1000 iterations, and posterior node probabilities were based on 2 million generations and estimated sample sizes over 100 (implemented in MrBayes) [18]. 

## 4. Conclusions

Mammalian reservoirs of zoonotic viruses typically do not display host restrictions within a given taxonomic order. Also, infection is usually chronic, persistent and subclinical. For example, rodents of multiple genera and species, belonging to four subfamilies in the order Rodentia, serve as reservoirs of hantaviruses in Eurasia, Africa and the Americas and do not exhibit clinical disease or survival disadvantage. In addition, recently, hantaviruses exhibiting far greater genetic diversity have been detected in healthy-appearing shrews and moles representing many genera in six subfamilies within the order Soricomorpha in Eurasia, Africa and North America. Similarly, as mentioned earlier, bat species belonging to both suborders of Chiroptera host hantaviruses without evidence of apparent disease. However, some might contend that the low prevalence of hantavirus RNA in a few bat species, and the absence of hantavirus infection in the majority of bat species analyzed to date, would argue against a long-standing hantavirus-reservoir host relationship, and instead support spillover or host switching. That is, the gleaning feeding behavior of some bats, such as *Nycteris*, presents the possibility of acquired infection from excreta of well-established terrestrial reservoirs of hantaviruses. However, this seems highly improbable because bat-borne hantaviruses are among the most genetically diverse described to date. 

With the discovery of divergent hantavirus lineages in three taxonomic orders of placental mammals, there is renewed interest in investigating their genetic diversity, geographic distributions, and evolutionary dynamics [34,35]. Newfound knowledge that insectivorous bats harbor a distinctly divergent lineage of hantaviruses emphasizes the truly complex evolutionary origins and phylogeography of a group of viruses once thought to be restricted to rodents. At this point, it would not be surprising if hantaviruses are found in small mammals belonging to other taxonomic orders, such as Erinaceomorpha (hedgehogs) and even Afrosoricida (tenrecs). Such anticipated discoveries may provide additional insights into the dynamics of hantavirus transmission, potential reassortment of genomes, and molecular determinants of hantavirus pathogenicity. As importantly, a sizable expansion of the hantavirus sequence database would provide valuable tools for refining diagnostic tests and enhancing preparedness for future outbreaks caused by emerging hantaviruses.
